# Application of Magnetic Concentrator for Improvement in Rapid Temperature Cycling Technology

**DOI:** 10.3390/polym13010091

**Published:** 2020-12-28

**Authors:** Krzysztof Mrozek, Paweł Muszyński, Przemysław Poszwa

**Affiliations:** 1 Institute of Mechanical Engineering, Poznan University of Technology, Piotrowo 3, 61-138 Poznan, Poland; krzysztof.mrozek@put.poznan.pl (K.M.); pawel.muszynski@put.poznan.pl (P.M.); 2 Institute of Materials Technology, Poznan University of Technology, Piotrowo 3, 61-138 Poznan, Poland

**Keywords:** injection molding, induction heating, rapid temperature cycling (RTC)

## Abstract

The main method to improve the filling of the cavity by the polymer melt is to increase the mold temperature. Rapid temperature cycling (RTC) technologies have been used around the world for several years, improving the quality of injection molded parts with a slight extension of production time. The present work focuses on the application of induction heating technology in the injection molding process since it is the most effective and most intensively developing method of heating in modern RTC technologies. In this research, the application of the induction heating process for selected surfaces was investigated with particular emphasis on the dynamics of the process. The numerical simulations were used to study the influence of the number of coils, the distance between coils and cavity surface and the mold material was examined with and without the presence of a magnetic concentrator. According to the obtained results, several crucial conclusions were made: the efficiency of heating is directly correlated with the distance between the inductor and the mold surface, the presence of magnetic flux concentrator significantly improves the homogeneity of temperature distribution and heating efficiency, application of aluminum mold lowers the obtained surface temperature.

## 1. Introduction

Nowadays, polymeric materials are widely used in many industries, such as construction, medical, electrical, and automotive [1,2,3,4]. Currently, high requirements are being placed on plastic parts [5]. Consequently, constant improvements in injection molding technologies become a necessity. Modern trends in polymer processing result from the expectations to improve the quality of manufactured products with reduced manufacturing costs. The main goal is to reduce the thickness of the wall of the injection-molded part, because the thinner the wall, the shorter the cooling time can be achieved. Thus, the entire injection cycle shortens. Reducing wall thickness results in increased injection pressure, due to the reduction in the cross-section of the mold’s cavity of the flow path of the plastic [6]. A thin layer of polymer melt cools down rapidly, increasing the resistance of the flow and leads to freezing of the material before the complete filling of the cavity [7]. The increase of the surface temperature above the transition temperature of the polymer melt allows the manufacturing of long thin-walled parts. For this purpose, Rapid Temperature Cycling (RTC) technology was developed, where the surface of the mold is periodically heated [8,9,10,11].

The higher surface temperature can significantly increase the time of part cooling, especially when technologies such as steam heating or cartridge heating are used [10,11,12,13]. This is due to the fact that not only the surface but the whole mold is heated to increase the surface temperature. To solve this disadvantage of increased cooling time, new techniques are developed, such as conformal cooling and algorithms that provide better distribution of cooling channels or optimized heating procedure [13,14,15,16].

The application of induction heating for RTC technology has been thoroughly investigated over the last years because of the high heating rate and small heating volume—which leads to improvement of the process with a small increase of the cooling time. New solutions are developed for this technique for better control of the heating region and even higher heating rate [17,18,19].

Next to the improvement of polymer flow, the high surface temperature provides several benefits at the cost of a longer process cycle. Higher cavity surface temperature can improve the part stiffness and tensile strength [15] and the functional properties as well [20]. This can be also used to minimize the reduction in tensile strength in parts, where weld lines are present [21,22,23]. Besides, the high cavity surface temperature is commonly used to improve surface quality (removal of weld lines’ presence, obtaining high-gloss surface, better replication of microstructure of the cavity surface) [6,11,23,24,25,26,27,28].

To increase the intensity of the induction heating, magnetic concentrators are used. Magnetic concentrators increase the effectiveness of the heating process by concentrating magnetic flux lines on the most important heating areas (magnetic field control), thereby significantly reducing the duration of the heating. Besides, more heating power is transferred to the selected area of the heated element from the coil with the concentrator reducing energy consumption [10]. Concentrators are made of magnetic powders and dielectric binders pressed under high pressure and heat treated. These materials are characterized by low electrical conductivity, high permeability, and low magnetic losses [29].

Numerical simulations can be used to examine complex systems [30,31,32], such as the induction heating in injection molds [33]. The main aim of this research was to use numerical simulations to investigate the influence of the presence of a magnetic concentrator along with inductor configuration on the heating process in the injection molding process. This article thoroughly analyzes the correlation between the heating time and the average, maximum temperature and its distribution to show the complexity of the heating process and to provide advice on optimization the heating phase. The obtained results showed that the distance between the inductor and the heated surface has a very strong impact on heating efficiency and that the presence of a magnetic concentrator can reduce heating time by more than 50%, especially in the case of a bigger distance between the inductor and heated surface.

## 2. Materials and Methods

In the present article, simulation studies of selective induction heating of injection molds were conducted. The simulation analysis was carried out by using the Finite Element Method (FEM) implemented in the QuickField 6.3.1 (Tera Analysis Ltd., Knasterhovvej 21, DK-5700 Svendborg, Denmark) package as a coupled problem. QuickField is able to import loads (distributed sources) calculated in a certain problem into the issue of another type-heat transfer caused by Joule heat generated in the AC magnetic problem. All of the tests were performed in 2D (XY) space in AC Magnetics modules (electromagnetic analysis) to determine the current density on the surface of the metal insert, followed by Transient Heat Transfer (thermal non-stationary analysis) to determine cavity surface temperature as a function of time.

In the present article, the numerical investigation was focused exclusively on the heating part of the RTC technology.

The AC magnetic problem is formulated as a partial differential equation for the complex amplitude of magnetic vector potential A (B = curl A, B—magnetic flux density vector). It is assumed that the flux density lies in the plane of the model (*xy* or *zr*), while the vector of electric current density **j** and the vector potential A are orthogonal to it. Only *jz* and *Az* in the planar case are not equal to zero. We will simply denote them *j* and *A.* The equation for the planar case is:

$$\frac{\partial }{\partial x}\left(\frac{1}{\mu_{y}}\frac{\partial A}{\partial x}\right)+\frac{\partial }{\partial y}\left(\frac{1}{\mu_{x}}\frac{\partial A}{\partial y}\right)-i\omega \sigma A=\;-j_{0}$$

where electric conductivity *σ* and components of magnetic permeability tensor *μx* and *μy* are constant in each model block. The source current density *j*0 is assumed to be constant in each block model in the planar case.

In this case, Dirichlet’s conditions were specified to solve the problem. The vector magnetic potential *A*_0_ at the edge of the model was set to 0.

For the heat transfer problem, the following set of local and integral physical quantities was calculated:

- Temperature *T*;
- Vector of the heat flux density F = −λ grad *T*
  $$\mathrm{Fx}=-{\;\lambda }_{x}\frac{\partial T}{\partial x},\;\mathrm{Fy}=\;-{\;\lambda }_{y}\frac{\partial T}{\partial y}$$
- Heat flux through an arbitrary closed or unclosed surface:

$$\Phi =\int_{S}^{}\left(F\cdot n\right)ds$$

where n denotes the unit vector of normal to the surface.

The analyzes included materials presented in Table 1.

**Figure 1:**
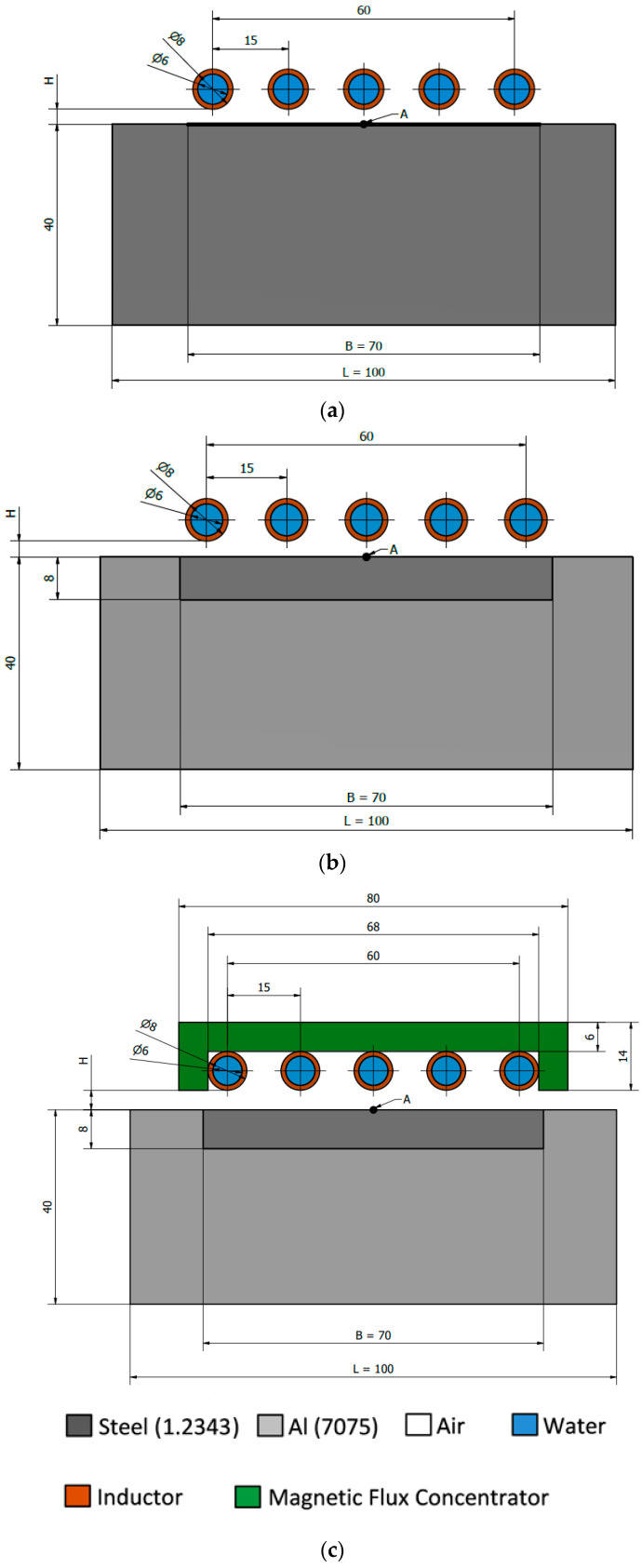
Geometries of (**a**) the steel block (P1); (**b**) the steel insert in the aluminum block (P2); (**c**) the steel insert in the aluminum block with the use of the magnetic flux concentrator (P3).

Figure 1a–c presents the analyzed geometries—steel block (P1), steel insert in the aluminum block (P2), steel insert in the aluminum block with the use of the magnetic flux concentrator (P3). Variants P2 and P3 involve bimetallic configuration—the body is made of aluminum alloy 7075, while the insert is made of 1.2343 steel. This solution was used to focus the magnetic field lines only on the steel insert, and thus heating only its surface. Aluminum, as a paramagnetic material, is not susceptible to the phenomenon of induction and the induced eddy currents, therefore it will remain “cold”. This phenomenon is very desirable for controlling the temperature of injection molds since only the cavity surface is being heated, which significantly reduces the volume of material for cooling.

**Figure 2:**
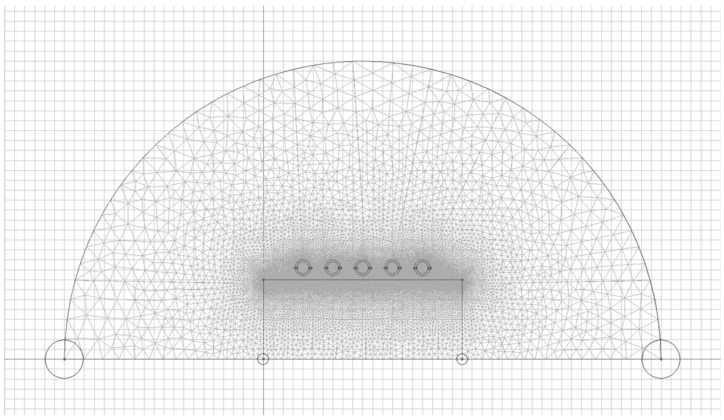
Discretization of the investigated system.

Based on the prepared geometries, a triangle mesh was generated (Figure 2). The density of the mesh and the size of the finite elements were adapted to the considered issue—the increased density of the mesh on the analyzed insert’s surface due to the skin effect.

The QuickField software can refine the mesh adaptively basing on the results of the previously solved problem (process also known as H-refinement). This capability practically eliminates the need for manual mesh control, allowing automatic mesh density adjustment in regions with a very inhomogeneous field.

The adjusted mesh spacing is calculated in every mesh node basing on the variation of the energy density in the node’s vicinity, which is proven to be the most reliable estimation of the error distribution. Although it is not possible to guarantee the precision, the refined mesh reaches the optimum size providing the best precision for the entire given number of mesh nodes.

The smooth mesh is generated with adjusted spacing distribution, but manually set mesh controls are also honored as top limits, guaranteeing that the mesh spacing will never exceed the user-specified values. Even with a fully automatic initial mesh, one h-refinement iteration is sufficient for most of the problems.

To create the mesh, an automatic mesh refining module was used, which is included in the QuickField software. The other areas were discretized based on the gradient of the length of elementary segments of individual edges and the obtained total node count was equal to 441,838 nodes. Table 2 presents the basic parameters in the inductor and the output data for simulation.

In the present work, the influence of the following geometrical factors was analyzed:

- the geometry of the heated mold: P1—steel block; P2—aluminum block + steel insert, P3—aluminum block + steel insert + magnetic concentrator
- coil distance from the heated surface H in the range from 1 mm to 4 mm (range selected so that the heating is both technologically possible and efficient): P1, P2, P3
- presence of the concentrator, number of coils: P2, P3
- number of coils from 2 to 5: P2, P3
- insert material (Table 1): P2

The simulations were analyzed as a function of time and as a function of the X coordinate directed along the heated surface.

**Figure 3:**
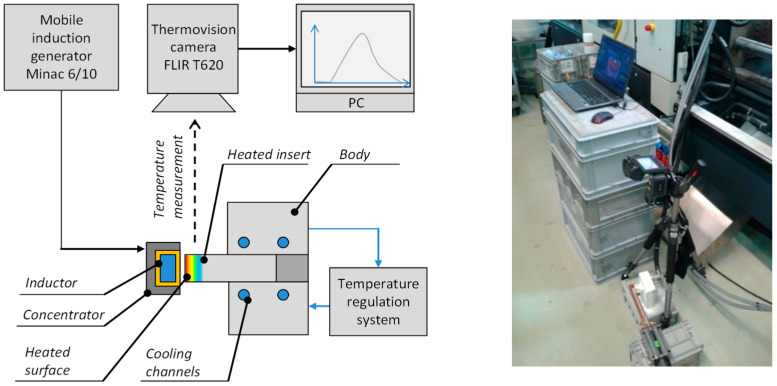
The experimental validation setup.

The numerical simulations were validated with the experimental setup that contained: induction generator EFD Minac 6/10, a coil with a magnetic concentrator, thermal imaging camera FLIR T620, temperature regulation system PSG, and injection mold block with changeable inserts. The precise description of the experiment can be found at [7], while Figure 3 shows a scheme and a photograph of the test stand.

## 3. Results

The analysis was focused on the temperature distribution. In this work, the temperature distribution in the block was analyzed along with the cavity surface temperature and change of temperature at the center of the mold.

**Figure 4:**
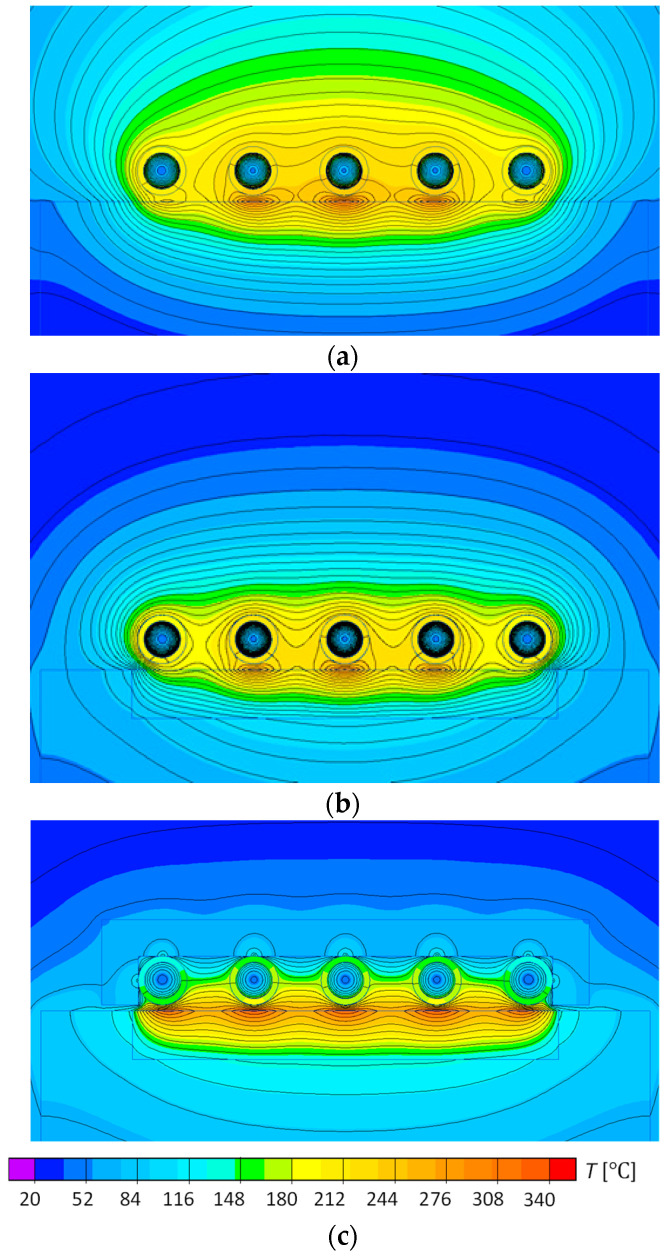
The temperature inside the block distribution after 10 s of heating for geometries (**a**) P1, (**b**) P2, and (**c**) P3.

Figure 4 presents the distribution of the temperature inside of the mold block for geometry P1–P3. This distribution is important in the case of heat removal, the presence of aluminum block (P2) leads to stronger heat transfer to the inside of the block and smaller temperatures at the surface because of the higher thermal conductivity of aluminum. By the application of aluminum block the heat removal would be more intensive than for steel block, but reaching the specific surface temperature would take more time. Application of magnetic flux concentrator (P3) led to more uniform temperature distribution at steel insert, the higher temperature at the surface of the mold and in the block. This means that the heating was overall more efficient than without a magnetic flux concentrator.

**Figure 5:**
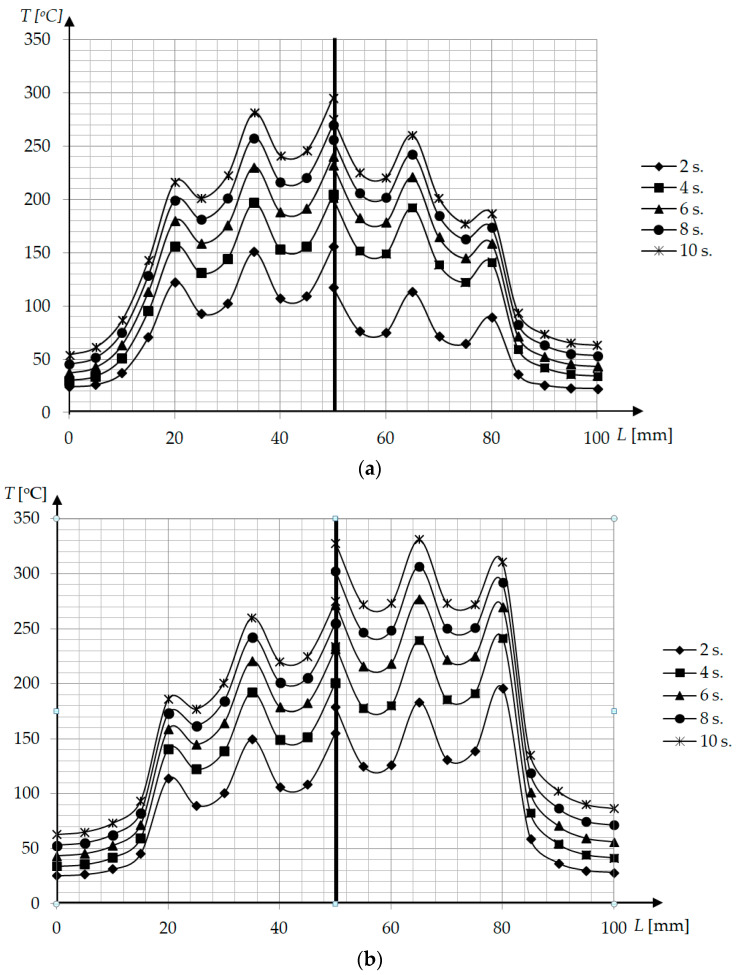
Cavity surface temperature distribution during induction heating at distance H = 1 mm for (**a**) P1 (left) and P2 (right) geometry, (**b**) P2 (left) and P3 (right) geometry.

The temperature distribution on the cavity surface for geometries P1–P3 in the function of time is presented in Figure 5 The highest temperature was observed under the central coil and the temperature fluctuates at the distance L. For extreme areas where coils are not present the temperature was lower, which indicates that the heating was so intense that the heat did not manage to dissipate significantly around the block. This confirms that the selected dimensions of the test geometry are well-chosen for the real injection molding process.

The temperature distribution was similar for geometries P1 and P2, but the higher surface temperature was observed for steel block (P1) than for aluminum block (P2) due to the higher thermal conductivity of aluminum (Figure 5). When a magnetic field concentrator is present (P3), the temperature surface distribution is significantly more uniform, along with higher temperatures obtained for consecutive time steps.

**Figure 6:**
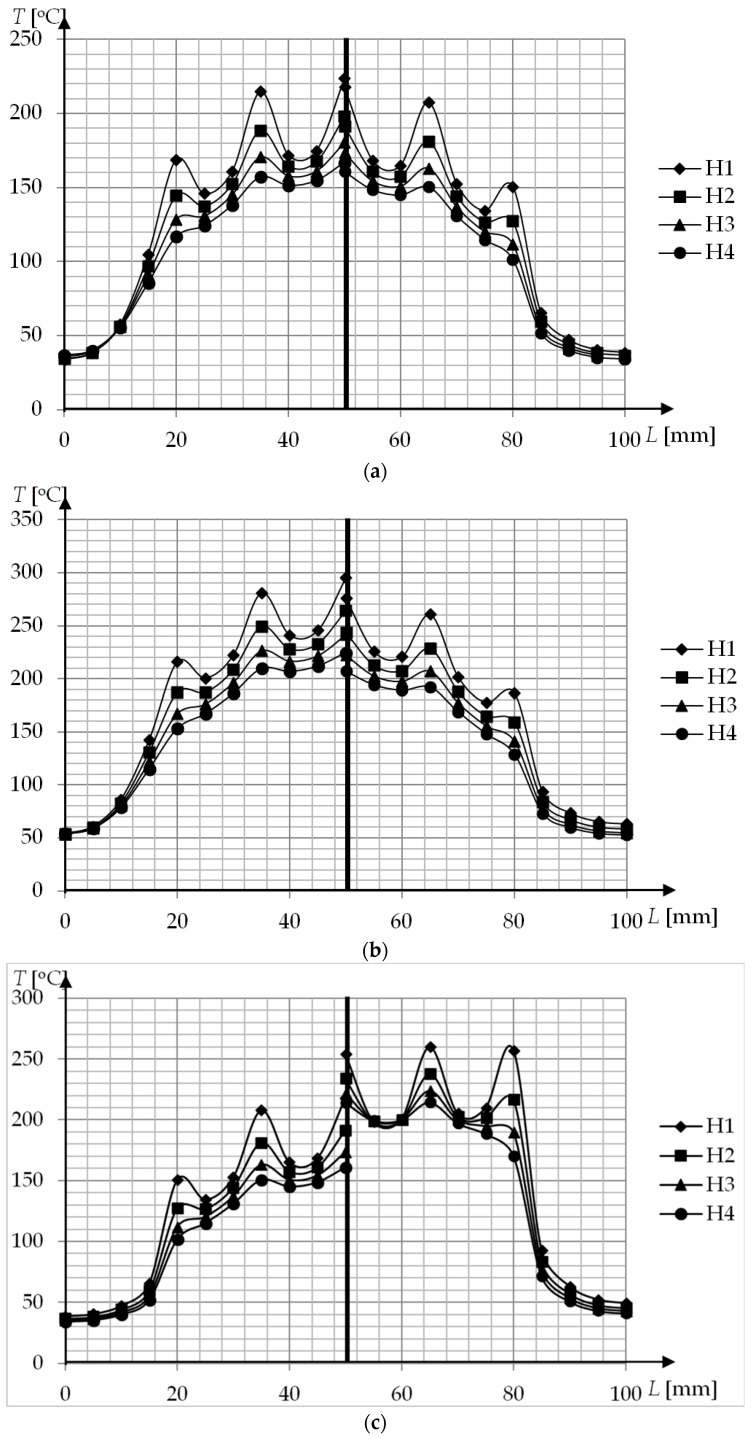

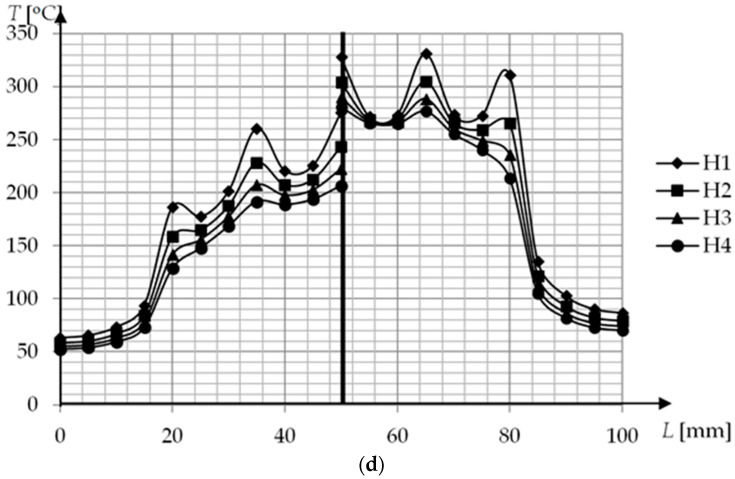
Cavity surface temperature distribution after induction heating for different distances H (**a**) t = 5 s, P1—left, P2—right; (**b**) t = 10 s, P1—left, P2—right; (**c**) t = 5 s, P2—left, P3—right; (**d**) t = 10 s, P2—left, P3—right.

The temperature distribution at the cavity surface for geometries P1–P3 in the function of the distance between inductor and surface (for t = 5 s and t = 10 s) is presented in Figure 6. The cavity surface has a very similar temperature distribution for t = 5 s and 5 = 10 s. The only difference is present in the temperature values. With the increase of the distance between the inductor and the surface H, the differences are much more visible. The increase of the H leads to smaller average temperatures and a more uniform temperature distribution, which is important in injection molding. For geometries P1–P3 the maxima at the external coils become flat when H is 3–4 mm.

**Figure 7:**
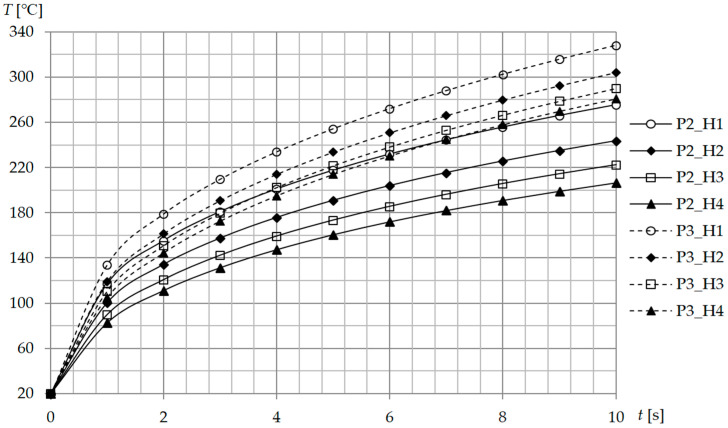
Temperature at point A (center of the cavity surface) in the function of time t for different geometries and distances between mold and inductor.

The temperature value at the center of the mold (point A) was measured in function of time for geometries P2 and P3 and different distances H. The results are presented in Figure 7. The most significant temperature change is present at the first second where temperature rises by 60–120 °C (depends on the H and presence of magnetic field concentrator). After the first second, the temperature change rate $\dot{T}$ gradually drops and after 3 s becomes almost linear. 

The application of a magnetic field concentrator leads to a situation where longer heating times the temperature is higher for H4 than for P2_H1 where a magnetic field concentrator is not present.

**Figure 8:**
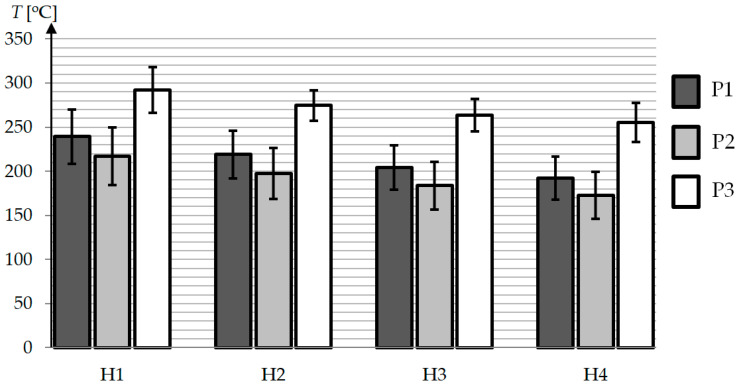
Cavity surface temperature for different geometries and distances between mold and inductor.

There were no significant differences in the shape of the curves between the individual variants P1–P3, the differences were only observed in the temperature values. To assess the heating efficiency, a column graph was prepared (Figure 8), which shows the average temperature of the heated area (an area equal to the width of the insert B marked in Figure 1a–c equal to 70 mm). The size of the standard deviation, which is a measure of temperature heterogeneity in the studied area, is marked on the individual columns by error bars. Based on the graph, we can see in P2 that the average temperature is around 25 °C lower than in P1. However, the use of the concentrator (P3) had a significant impact on the intensity of heating, increasing the average surface temperature by about 70 °C in comparison to the setup without the concentrator (P2).

**Figure 9:**
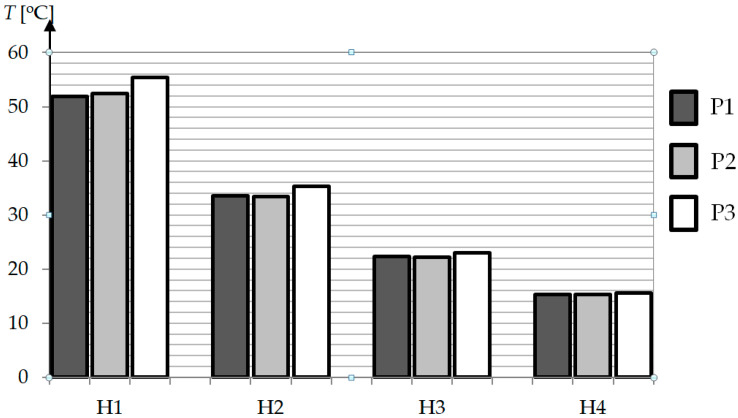
The temperature difference between points A and C for different geometries and distances between mold and inductor after t = 10 s.

Increasing the distance between the inductor and the cavity surface reduced the average temperature, wherewith each subsequent millimeter the decrease was smaller (Figure 9). It is worth noting that as the distance increased, the differences in average temperature values for P1–P3 did not change significantly. As the distance increased, a difference of ca. 10 °C was observed between 1 and 4 mm for P1 and P2, and only by 4 °C for P3. It should be mentioned that the smallest standard deviation was present for P3 and the largest for P1—even if the differences presented in Figure 9 are greater for P3, the height of subsequent peaks is more equal for P3.

**Figure 10:**
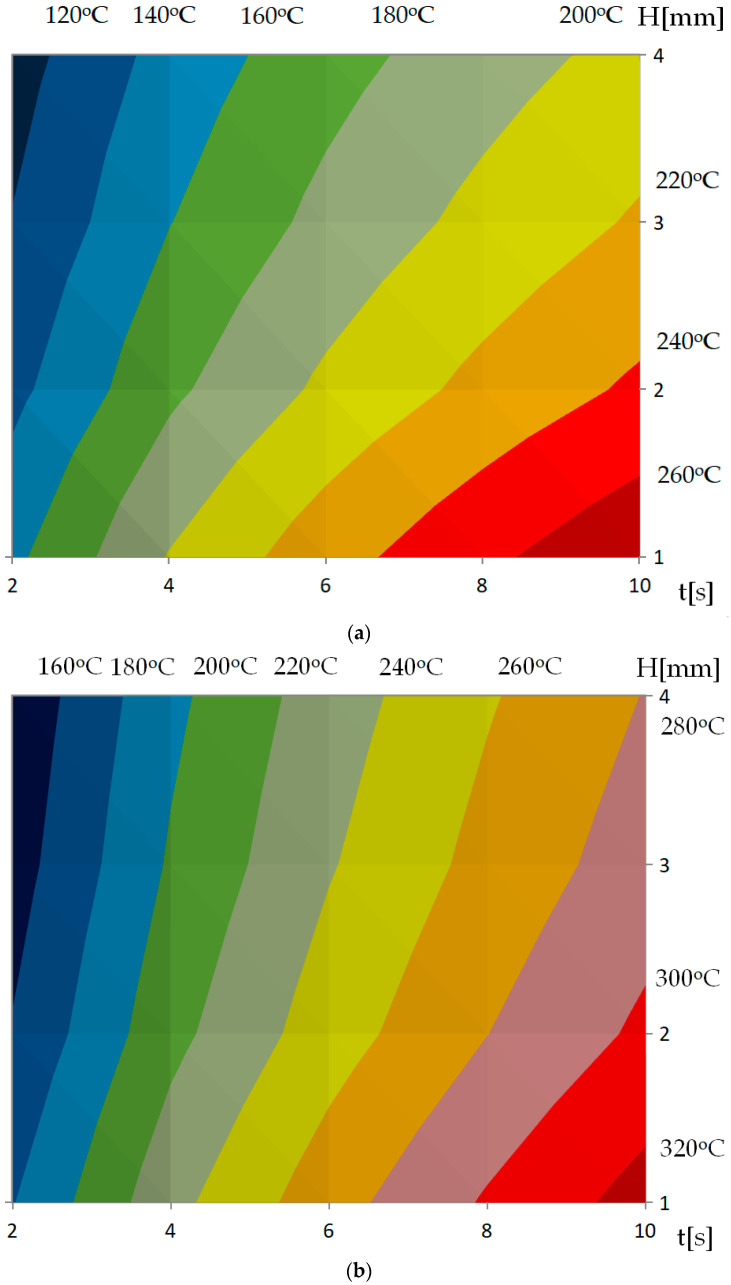
Temperature at point A (center of the cavity surface) in the function of time *t* for different distances between mold and inductor (equal to maximum surface temperature) and for (**a**) P2, (**b**) P3.

Maximum temperature is presented in Figure 10 in function of heating time t and distance between inductor and cavity surface H. This kind of contour plots can be easily used to optimize the heating process as the expected value is presented between the individual bands. For proper use of this diagrams, it is necessary to take into account the information about temperature heterogeneity in order to select conditions that will ensure acceptable temperature distribution for RTC technology.

**Figure 11:**
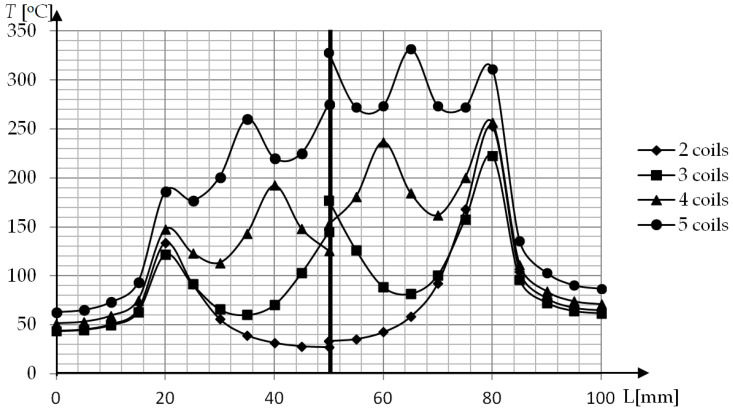
Cavity surface temperature distribution after induction heating (t = 10 s) for a different number of coils. P2—left, P3—right.

**Figure 12:**
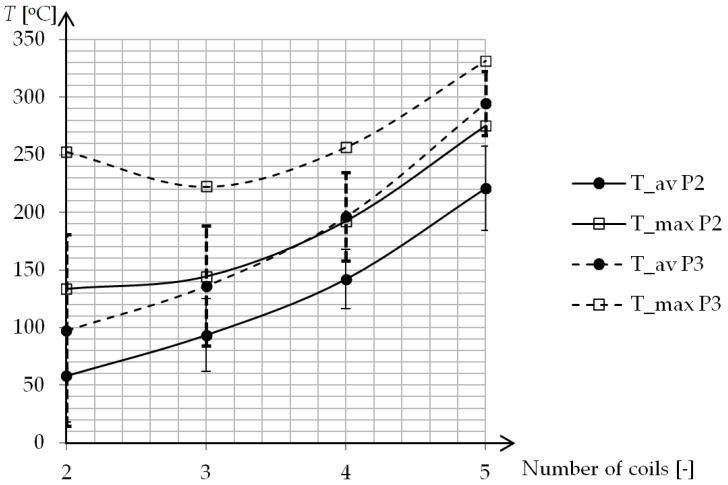
Average (T_av) and maximum (T_max) cavity surface temperature for different geometries.

In the study, the influence of a different number of coils on cavity surface temperature distribution was also investigated. In the research geometry, P2 and P3 were simulated where the number of coils changed from 2 to 5 (Figure 11). The least significant difference in temperature for both geometries is present for external coils. For P2 geometry the maxima gradually rise to the center of the mold in all cases. In the case of P3 geometry, there is a surprisingly higher temperature in the external coils than at the center coil, when there are 3 and 4 coils in the external coils. The application of a magnetic field concentrator leads to smaller differences between maxima, but higher heterogeneity of temperature distribution (Figure 12). The increase of average temperature for P2 and P3 have a very similar shape with the shift equal to 40–50 °C.

**Figure 13:**
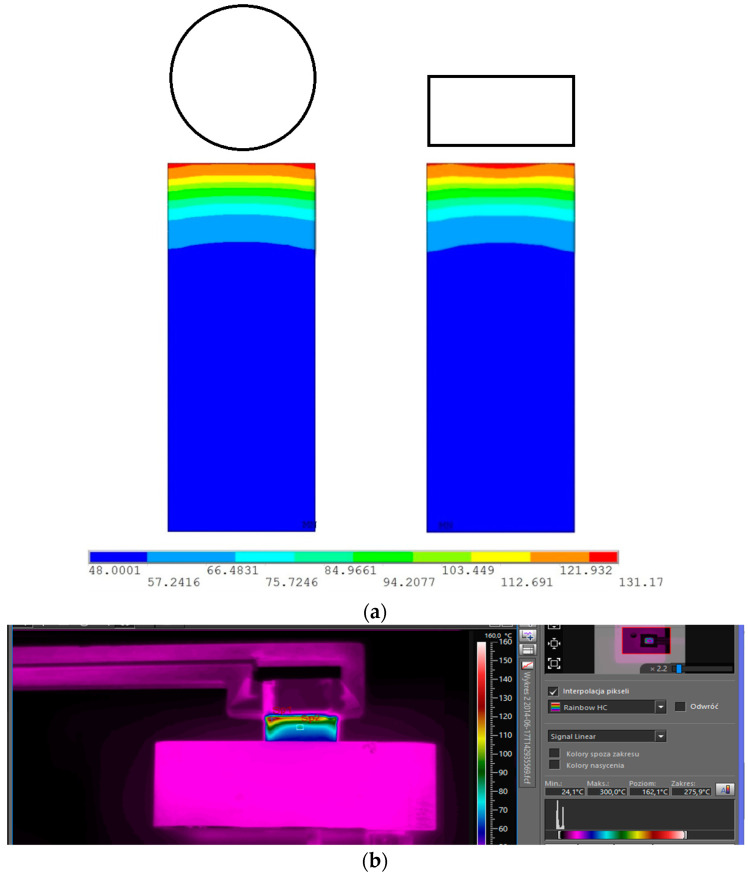
(**a**) The comparison of temperature profile of heated insert with different shapes of inductors heating time was equal to 2.5 s and the distance between inductor and surface was equal to 1 mm. (**b**) Measurement of the surface temperature heated with an induction coil.

The numerical results were validated with the experiment where a slightly different coil shape was used. In this article, the annular coils are used, whereas in the experimental setup the hollow rectangular profile was used. It was verified that the influence of inductor’s cross-section shape is negligible in terms of heating efficiency. The numerical simulations were verified the thermal measurements with thermal camera. The results of the performed verifications are presented in Figure 13.

**Figure 14:**
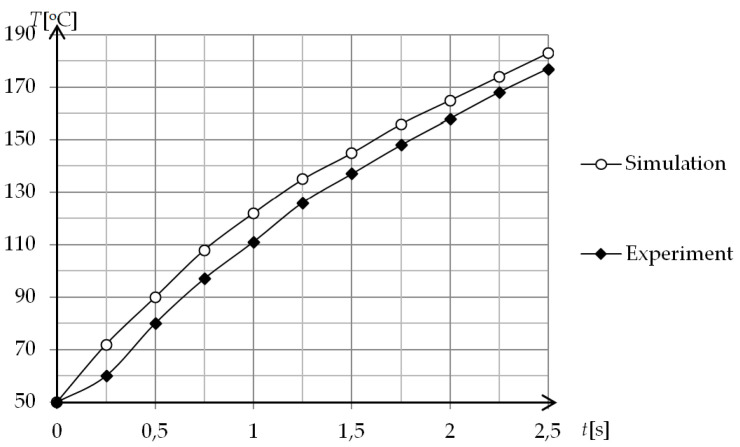
Comparison of numerical and experimental results for induction heating with hollow rectangular coil.

Both numerical and experimental results from the hollow rectangular are presented in Figure 14. According to the obtained results, there is a slight difference between the numerical and experimental results. The numerical results show a small overrun of surface temperature (about 10 °C) for a specific time.

The diagram above shows a comparison of the heating process determined in a simulation and experimental way for the coil at a distance of 1 mm from the heated surface. The characteristics of both processes are very similar. The temperatures recorded with a thermal vision camera are lower by 9–12 °C depending on time. The differences may result from the material properties of the heated insert. The theoretical model excluded parameters depending on mechanical and heat treatment, and these aspects influence the heating process. The second reason for the discrepancy of results is the lack of consideration of the mixed convection phenomenon in the theoretical model. During the simulation studies, the air movements that occur during experimental studies were not modelled. The third reason is the difference between the number of dimensions included in simulation (2D) and experiment (3D).

## 4. Discussion

In this research, the efficiency of induction heating of injection molds was investigated. Based on the results of the simulation, the following conclusions and observations were formulated:

- The dynamics of the induction heating process closely depend on the distance of the inductor from the heated surface, the number of coils, the distance between the coils, the presence of the concentrator, and the material used for the inserts.
- Induction heating of selected cavity surfaces enables efficient and energy-saving injection process with rapid temperature changes of the injection mold where with the presence of the magnetic concentrator the temperature rise of 200 °C can be achieved in 3–4 s. Without the magnetic concentrator, the time of heating can last even 9 s.
- The distribution of the temperature on the surface of the heated block was similar for all tested geometries. The difference was visible at maximum and average temperatures, as well as uniformity of heating. As the average temperature increased, an increase in dispersion measured by standard deviation was observed.
- During testing the influence of the inductor distance from the front of the heated surface on process efficiency, the highest temperature was recorded for the smallest of the analyzed distances—1 mm. In turn, the most uniform temperature distribution on the surface was obtained in the case of a gap of 4 mm (with the increase in distance the average temperature of the heated surface and the unevenness of heating decreased).
- There were higher average temperatures observed for the solid insert (P1) than for the bimetallic insert (P2) which may mean that a larger volume of ferromagnetic material has a positive effect on temperature increase.
- The construction of the bimetallic mold allows heating only those surfaces that are responsible for molding the pieces in the injection process (mold insert made of ferromagnetic material, mold body made of paramagnetic material).
- The temperature change was measured as a function of the heating time, where the largest increase in temperature at the point was observed in the first second. In subsequent seconds the temperature increase is significantly smaller and decreases with each second, which is related to the phenomenon of heat conduction. The temperature increase is present up to the Curie temperature, where the induction heating process is stopped.
- The number of coils has a significant impact on both the average and maximum heating temperatures. An irregular relationship between the maximum temperature and the number of coils was observed. It turns out that for 2 coils a higher maximum temperature was obtained than for 3 coils.
- The use of magnetic field concentrators significantly affects the efficiency of the heating process and allows for obtaining much higher temperatures compared to the inductors without concentrators.
- The contour plots can be used to optimize process parameters to obtain proper temperature distribution at the cavity surface.

## Figures and Tables

**Table 1:** Material properties used in the research.

Material	Steel1.2343	Aluminum 7075	Fluxtrol A	Copper	Water	Air
Permeability μ_r_	55	1	130	1	1	1
Electrical conductivity σ $\left[\frac{S}{m}\right]$	10^7^	3 × 10^7^	5 × 10^−5^	5.6 × 10^7^	2 × 10^−4^	5 × 10^−15^
Thermal conductivity *K* $\left[\frac{W}{\mathrm{mK}}\right]$	45	150	23	380	0.58	0.025
Density ρ $\left[\frac{g}{{\mathrm{cm}}^{3}}\right]$	7.8	2.7	6.6	8.7	1	0.001
Specific heat *C* $\left[\frac{J}{\mathrm{kgK}}\right]$	460	850	430	380	4190	1005

**Table 2:** Input data for simulation research.

Parameter	Value
Current *I* [A]:	1420
Initial temperature $T_{0}$ [°C]:	50
Frequency ν [kHz]:	22
Heating time *t* [s]:	10
Distance from cavity surface *H* [mm]	1
Number of coils *n* [-]	5
Basic geometry *P* [-]	P2 type
Length of measured distance [mm]	70 total, symmetrically from the center of the part

## Data Availability

The data presented in this study are available on request from the corresponding author.
